# Supplementary material for: An alignment-free method for phylogeny estimation using maximum likelihood
Source: BMC Bioinformatics. 2025 Mar 7;26:77. doi: 10.1186/s12859-025-06080-w (PMC11887328; doi:10.1186/s12859-025-06080-w)
Supplement: Supplementary file 1 — An alignment-free method for phylogeny estimation using maximum likelihood” contains all supplementary tables and figures. [file 12859_2025_6080_MOESM1_ESM.pdf]

Supplementary information: an alignment-free method for phylogeny estimation using maximum likelihood

**Methods**	**Datasets**						
	**29 E.coli**	**25 Fish**	**14 Plant**	**27 E.coil**	**8 Yersinia**	**7 Primates**	**Drosophila**
FFP	0.23	0.09	0.18	0.21	0.8	**0**	0.27
co-phylog	0.12	0.09	**0.09**	**0.08**	0.8	0.5	-
mash	0.15	**0.05**	**0.09**	0.12	0.8	0.25/**0**	0.18
Skmer	0.15	0.09	0.18	0.17	0.8	0.25/0.25	**0.09**
FSWM/Read-SpaM	0.12	**0.05**	0.27	0.17	1	**0/0**	0.18
PEAFOWL	0.23	**0.05**	0.36	0.17	1/**0**	**0**	**0.09**
andi	0.08	0.18	0.82	**0.08**	0.8	**0/0**	0.36
phylonium	**0.04**	0.14	0.64	**0.08**	0.8	**0/0**	**0.09**
Multi-SpaM	0.27	0.18	**0.09**	0.17	0.6	**0**	-
CAFE-cvtree	0.58	0.09	0.27	0.5	**0**	0.75/0.25	-
Median AF Project	0.54	0.09	0.64	0.5	0	-	-
Best AF Project	0.04	0.05	0.09	0.08	0	-	-

Table S1: **Comparison of normalized Robinson Foulds distances.** Comparison of normalized Robinson Foulds distance achieved by different alignment-free methods on real datasets. Minimum nRF values are in bold. For the 7 Primates column, values on the left and right side of oblique represent result obtained using Neighbour joining and UPGMA respectively. For the Yersinia dataset, result of *PEAFOWL* is written as 1/0 denoting nRF of 1 with -r parameter and 0 without.

kmer size	Datasets						
	**7 Primates**	**Drosophila**	**25 Fish**	**29 E.coli**	**14 Plant**	**8 Yersinia**	**27 E.coil**
9	**3615.96**	0	**2573.04**	79.7426	0	3.80495	77.3329
11	3392.6	0.742465	1698.89	1523.02	35.9579	279.236	1564.18
13	3278.95	2146.87	1520.86	2355.5	2340.57	698.685	2360.82
15	3251.11	**3241.13**	1465.35	2338.97	**3410.24**	842.232	2338.36
17	3224.27	2370.88	1409.93	2338.28	2456.5	846.749	2340.6
19	3179.1	2172.6	1383.88	2337.9	-	883.142	2370.37
21	3157.08	2101.94	1357.58	2310.44	-	963.98	2354.09
23	3145.58	2077.11	1330.91	**2378.96**	-	1030.61	2386.66
25	3107.85	2072.25	1317.63	2362.13	-	1040.54	**2419.14**
27	3093.87	2056.64	1321.92	2335.58	-	1094.62	2396.31
29	3092.59	2041.32	1303.63	2340.9	-	1054.41	2400.09
31	3067.9	2030.61	1305.73	2351.19	-	**1117.6**	2369.13
nRF	0	0.09	0.05	0.23	0.36	1	0.17

Table S2: **Canonical entropy values and normalized RF distances obtained by Peafowl.** Canonical entropy values and normalized RF distances obtained by PEAFOWL on different datasets. Maximum entropy values are highlighted in bold. Entropy values for 14 plant dataset are reported up to k-mer 17 to avoid resource exhaustion.

kmer size	Datasets						
	**7 Primates**	**Drosophila**	**25 Fish**	**29 E.coli**	**14 Plant**	**8 Yersinia**	**27 E.coil**
9	**3575.37**	0	**2315.76**	213.813	0	87.2463	207.321
11	3378.81	17.0652	1671.15	2560.25	141.889	2955.31	2574.37
13	3292.69	3244.44	1517.93	**2640.69**	3115.87	4400.54	**2645.71**
15	3259.07	**3469.01**	1449.79	2433.96	**3165.45**	4498.11	2473.8
17	3210.92	2210.08	1406.96	2370.21	2259.79	**4502.69**	2393.63
19	3181.13	2064.89	1377.99	2355.63	-	4491.7	2387.07
21	3165.69	2013.31	1346.19	2328.45	-	4502.33	2386.53
23	3137.06	2002.93	1331.23	2314.3	-	4496.69	2364.63
25	3116.49	1991.83	1332.8	2350.04	-	4459.67	2346.08
27	3091.58	1967.92	1320.05	2319.48	-	4463.77	2341.95
29	3086.13	1985.55	1303.31	2282.54	-	4467.69	2339.97
31	3071.44	1970.84	1300.85	2295.84	-	4431.96	2290.89
nRF	0	0.18	0.05	0.62	0.46	0	0.58

Table S3: **Non-canonical entropy values and normalized RF distances obtained by Peafowl.**Non canonical entropy values and normalized RF distances obtained by PEAFOWL on different datasets. Maximum entropy values are highlighted in bold. Entropy values for 14 plant dataset are reported up to k-mer 17 to avoid resource exhaustion.

**Dataset**	Total Time (hour)	k-mer Extraction time (%)	Matrix Construction and k-mer selection time (%)	Transpose time (%)	RAxML time (%)	# Matrix Rows (# k-mers)	# Matrix Columns (# species)
7 Primates	0.01	2.46	97.27	0.03	0.23	45,780	7
14 *Drosophila*	25.27	0.92	95.72	0.26	3.11	387,723,760	14
25 Fish	0.02	4.98	86.4	0.3	8.32	89,850	25
14 Plant (k=9 to 17)	8.30	10.69	76.34	0.86	12.11	419,875,520	14
29 *E. coli*	1.34	0.99	94.32	0.99	3.7	20,525,643	29
27 *E. coli*	1.64	0.77	95.6	0.74	2.89	20,703,383	27
8 *Yersinia*	0.47	1.46	97.76	0.13	0.64	9,851,968	8

Table S4: **Breakdown of runtime of various steps of Peafowl.** Breakdown of runtime of various steps on different datasets and the dimensions of the k-mer presence/absence matrix corresponding to $k_{entropy}$.

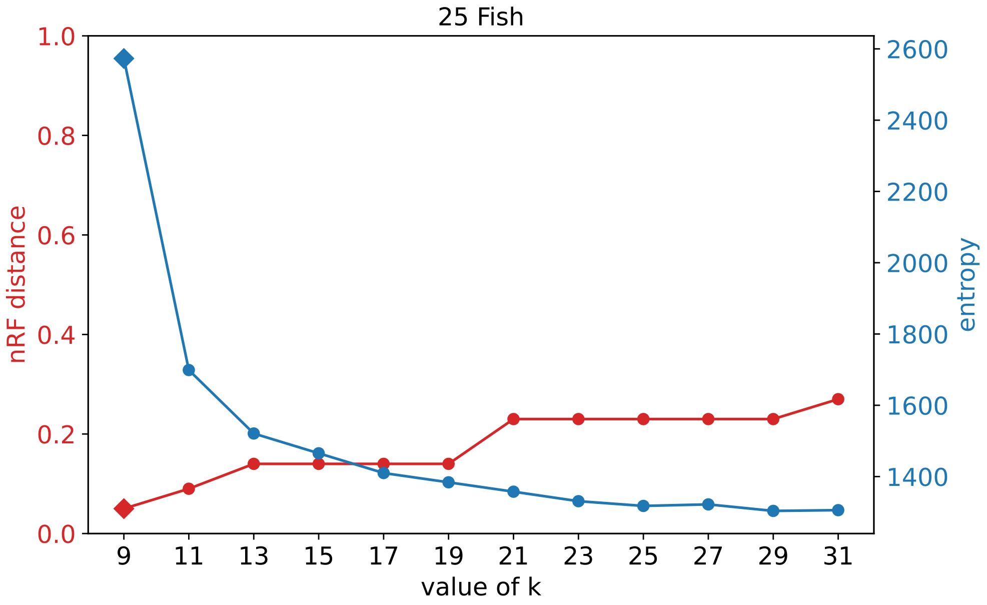

Figure S1: **Normalized Robinson Foulds distance and entropy vs. k-mer length for the 25-Fish dataset.** Variation of normalized Robinson Foulds distance and entropy with change in k-mer length for the 25-Fish dataset. Diamond shaped markers represent values corresponding to $k_{entropy}$ ($k = 9$).

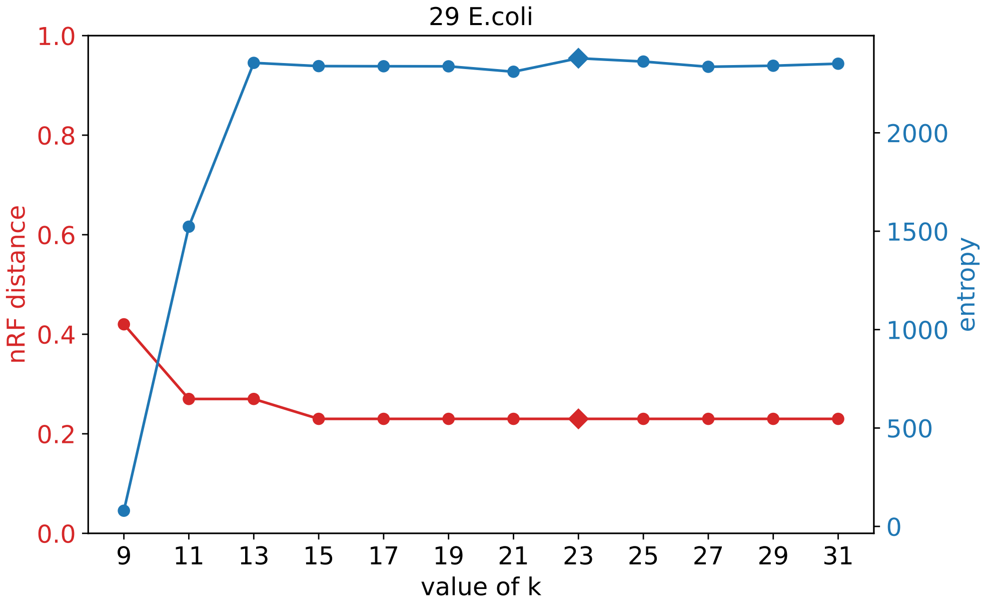

Figure S2: **Normalized Robinson Foulds distance and entropy vs. k-mer length for the 29 *E.coli* dataset.** Variation of normalized Robinson Foulds distance and entropy with change in k-mer length for the 29 *E.coli* dataset. Diamond shaped markers represent values corresponding to $k_{entropy}$ ($k = 23$).

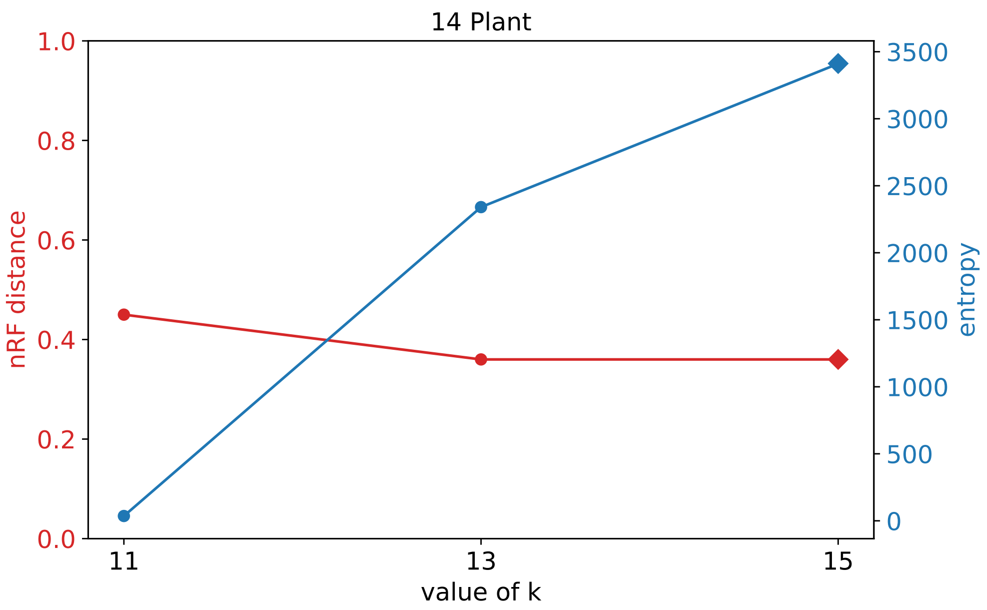

Figure S3: **Normalized Robinson Foulds distance and entropy vs. k-mer length for the 14 plant dataset.** Variation of normalized Robinson Foulds distance and entropy with change in k-mer length (k=11 to 15) for the 14 plant dataset. Diamond shaped markers represent values corresponding to $k_{entropy}$ (k = 15).

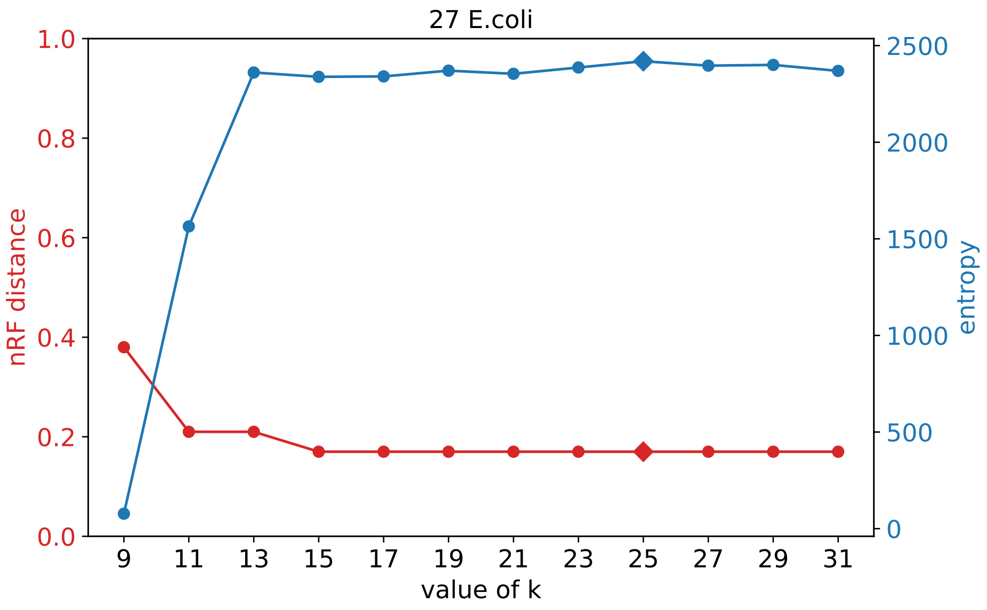

Figure S4: **Normalized Robinson Foulds distance and entropy vs. k-mer length for the 27 *E.coli* dataset.** Variation of normalized Robinson Foulds distance and entropy with change in k-mer length for the 27 *E.coli* dataset. Diamond shaped markers represent values corresponding to $k_{entropy}$ (k = 25).

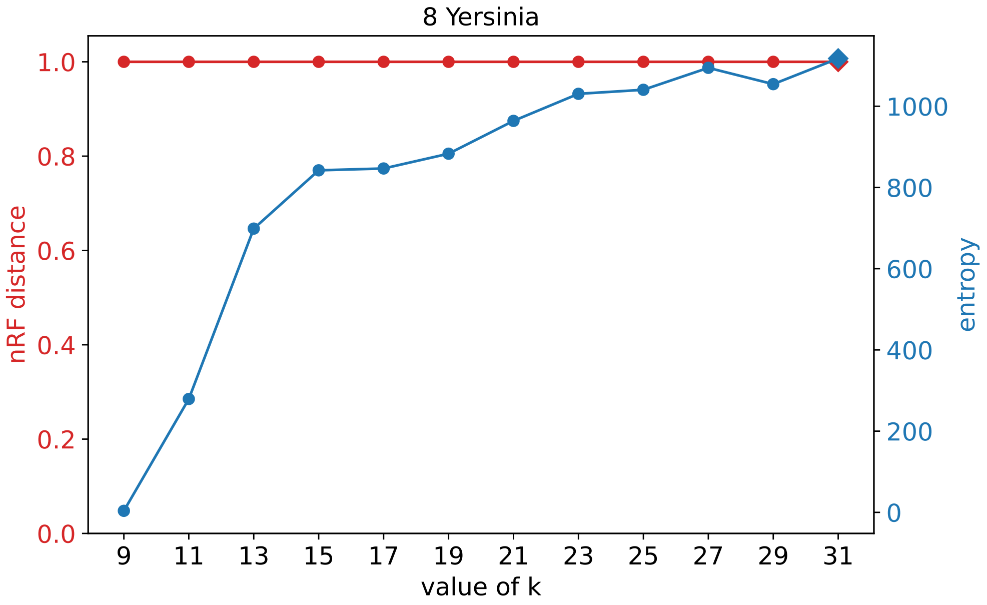

Figure S5: **Normalized Robinson Foulds distance and entropy vs. k-mer length for the 27 8-Yersinia dataset.** Variation of normalized Robinson Foulds distance and entropy with change in k-mer length for the 8-Yersinia dataset (with r parameter). Diamond shaped markers represent values corresponding to $k_{entropy}$ ($k = 31$).

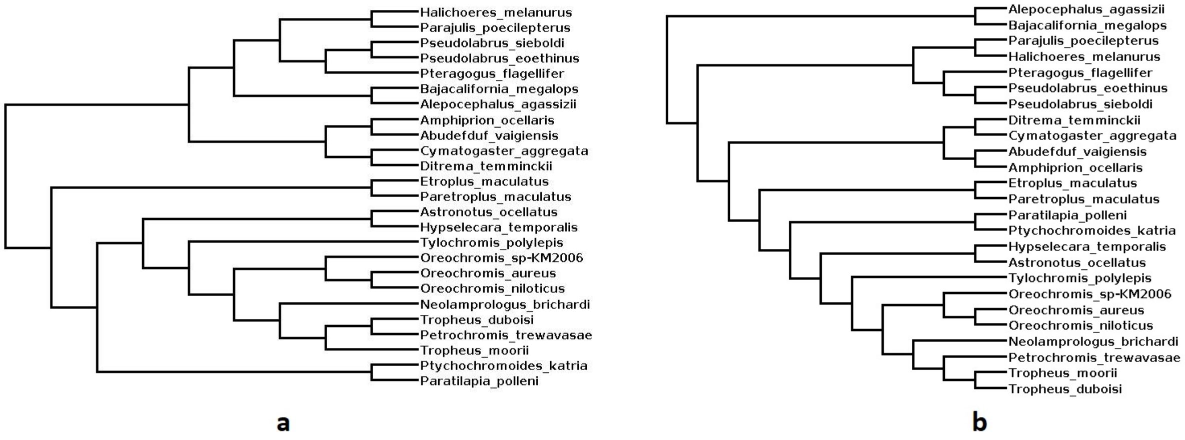

Figure S6: **Comparison of fish phylogenies. a.** Phylogeny generated by *PEAFOWL* and **b.** the benchmark tree on the Fish dataset.

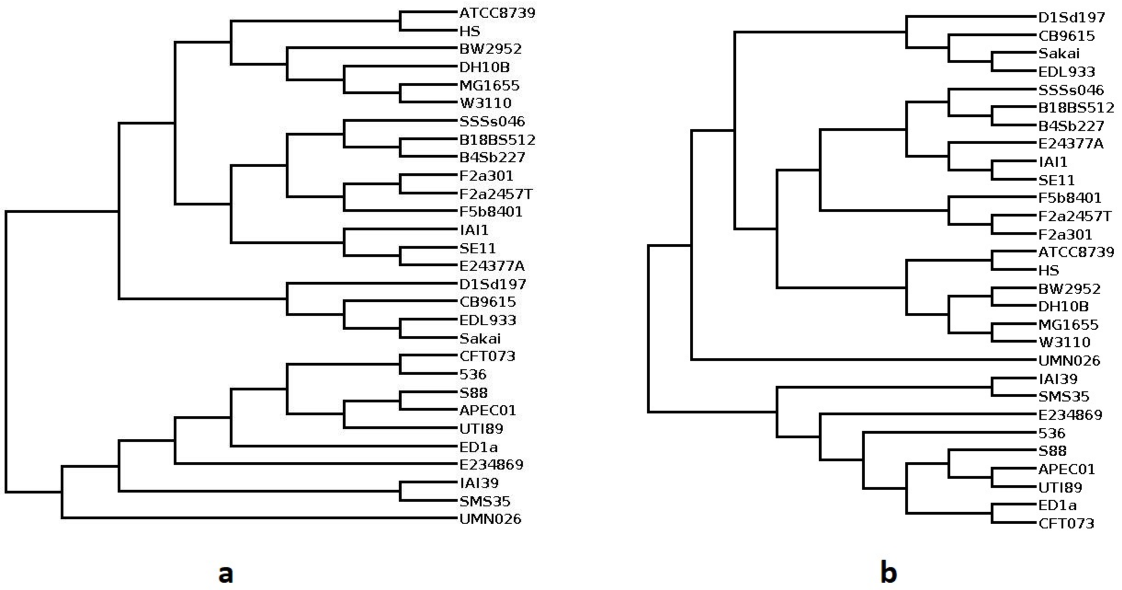

Figure S7: **Comparison of *E.coli* phylogenies. a.** Phylogeny generated by *PEAFOWL* and **b.** the benchmark tree on the 29 *E.coli* dataset.

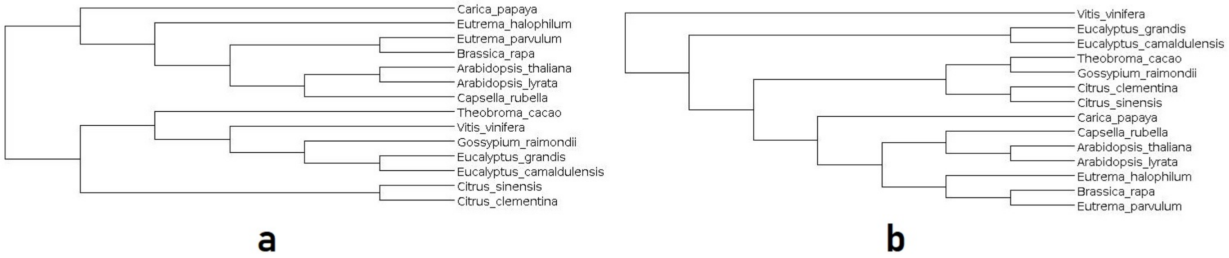

Figure S8: **Comparison of plant phylogenies. a.** Phylogeny generated by *PEAFOWL* and **b.** the benchmark tree on the 14 Plant dataset.

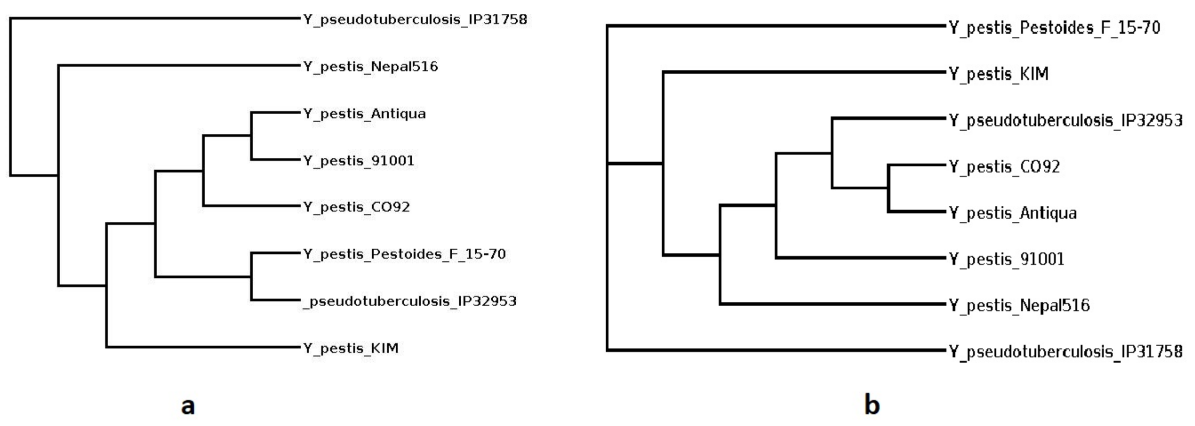

Figure S9: **Comparison of Yersinia phylogenies. a.** Phylogeny generated by *PEAFOWL* and **b.** the benchmark tree on the 8 Yersinia dataset.

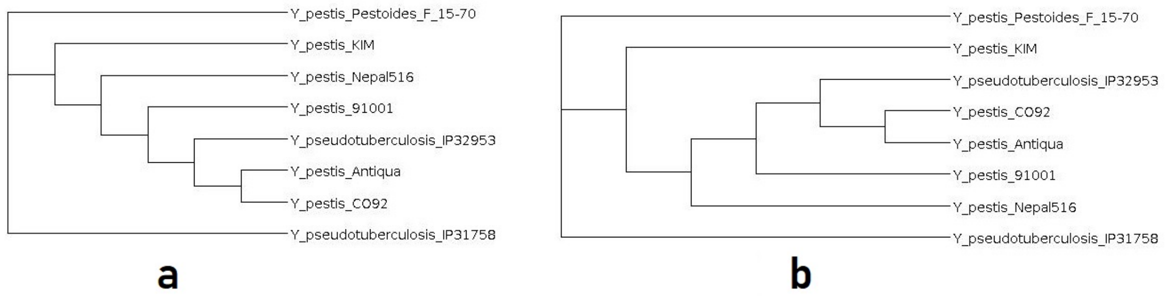

Figure S10: **Comparison of Yersinia phylogenies (without r parameter). a.** Phylogeny generated by *PEAFOWL* (without r parameter) and **b.** the benchmark tree on the 8 Yersinia dataset.

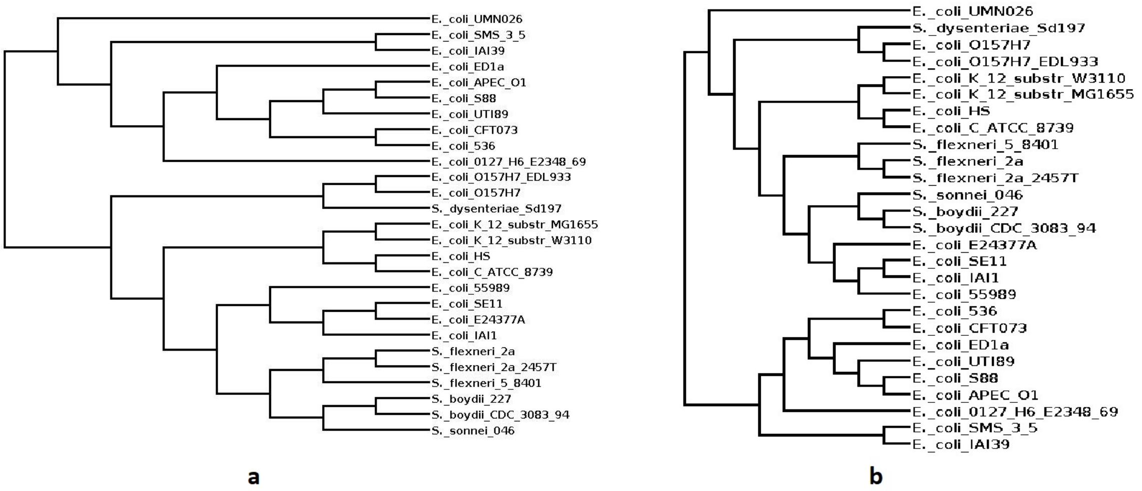

Figure S11: **Comparison of *E.coli* phylogenies. a.** Phylogeny generated by *PEAFOWL* and **b.** the benchmark tree on the 27 *E.coli* dataset.

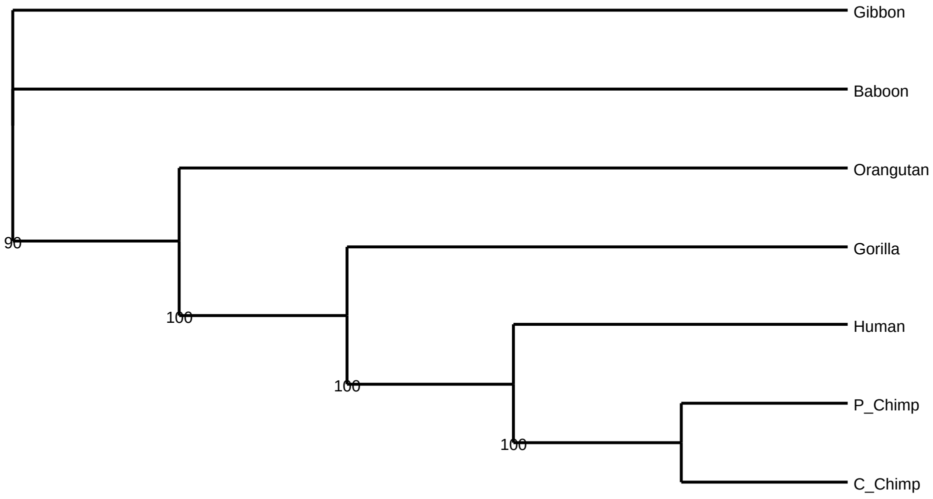

Figure S12: **Consensus tree from the bootstrap replicates for the 7 primates dataset**

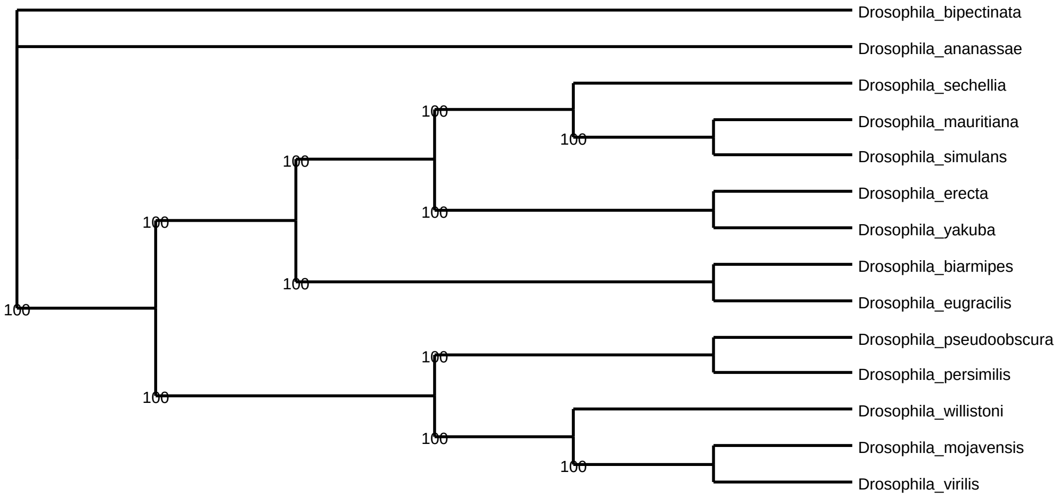

Figure S13: **Consensus tree from the bootstrap replicates for the *Drosophila* dataset**
